# Outcome of Patients Supported by Large Impella Systems After Re-implantation Due to Continued or Recurrent Need of Temporary Mechanical Circulatory Support

**DOI:** 10.3389/fcvm.2022.926389

**Published:** 2022-07-07

**Authors:** Yukiharu Sugimura, Sebastian Bauer, Moritz Benjamin Immohr, Arash Mehdiani, Philipp Rellecke, Ralf Westenfeld, Hug Aubin, Udo Boeken, Artur Lichtenberg, Payam Akhyari

**Affiliations:** ^1^Department of Cardiac Surgery and Research Group for Experimental Surgery, Medical Faculty and University Hospital Düsseldorf, Heinrich Heine University Hospital, Düsseldorf, Germany; ^2^Department of Cardiology, Angiology and Pulmonology, Medical Faculty and University Hospital Düsseldorf, Heinrich Heine University Hospital, Düsseldorf, Germany

**Keywords:** cardiogenic shock, Impella, complication, re-implantation, thrombosis

## Abstract

Despite the growing utilization of a large microaxial pump, i. e., Impella 5.0 or 5.5 (Abiomed Inc., Danvers, MA, USA) (Impella 5+) for patients with cardiogenic shock (CS), adverse events including the necessity of re-implantation have not been well discussed. In all 67 patients, in-hospital mortality was 52.2% (*n* = 35). Explantation of Impella 5+ was performed in 39 patients (58.2%), 22 of whom (32.8%) recovered under Impella 5+, and ten further patients (14.9%) survived after a successful transition to permanent mechanical circulatory support. Embolic events were considerable complications in each access. They occurred in the right arm after the removal of Impella 5+ *via* a subclavian artery (SA) (*n* = 3, 9.1%) or in the form of leg ischemia in patients with Impella 5+ *via* femoral artery (FA) (*n* = 2, 33.3%). Re-implantation was necessary for 10 patients (14.9%) due to 1) recurrent CS (*n* = 3), 2) pump thrombosis (*n* = 5), or 3) pump dislocation (*n* = 2), all of which were successfully performed *via* the same access route. In univariate analysis, FA access was a significant risk factor for Impella dysfunction compared to SA access (FA vs. SA, 42.9% vs. 9.8%, p < 0.05, odds ratio 6.88). No statistical difference of overall mortality was observed in patients with Impella 5+ re-implantation (*n* = 10) compared to patients with primary Impella 5+ support (*n* = 57) (80.0% (*n* = 8/10) vs. 47.4% (*n* = 27/57), *p* = 0.09). Our results suggested the acceptable clinical outcome of Impella 5+ despite a 15% re-implantation rate. Our observational data may merit further analysis of anticoagulation strategies, including risk stratification for embolic events.

## Introduction

In recent years, the percutaneous microaxial pump, i.e., Impella (Abiomed Inc., Danvers, MA, USA), has enabled antegrade flow support with unloading of the left ventricle (LV), which provides us with various therapy options to manage patients with cardiogenic shock (CS). Few previous studies have introduced some complications and adverse outcomes of Impella systems (1–6). In the field of heart failure surgery, our attention is directed to large microaxial pump catheters, i.e., Impella 5.0 or 5.5 (Impella 5+), since patients with fulminant CS often need larger Impella systems to mimic the hemodynamic status under left ventricular assist device (LVAD) support and to bridge the permanent mechanical circulatory support (MCS) as well as orthotopic heart transplantation (oHTX).

Despite a comprehensive utilization of Impella 5+ for patients with CS, the reports regarding adverse events of Impella 5+ are scarce (7, 8). Moreover, reports that focused on re-implantation of Impella 5+ and its impact on patient outcomes are yet missing. In this study, we report our experience with respect to adverse events and the clinical outcomes after Impella 5+ support to elucidate the effective postoperative management of Impella 5+ as temporary MCS. In particular, we analyze those cases with re-implantation of Impella 5+.

## Materials and Methods

### Ethics Committee Approval

The local ethics committee approved this retrospective study (Ref. 2020-1173).

### Study Population

**Figure 1:**
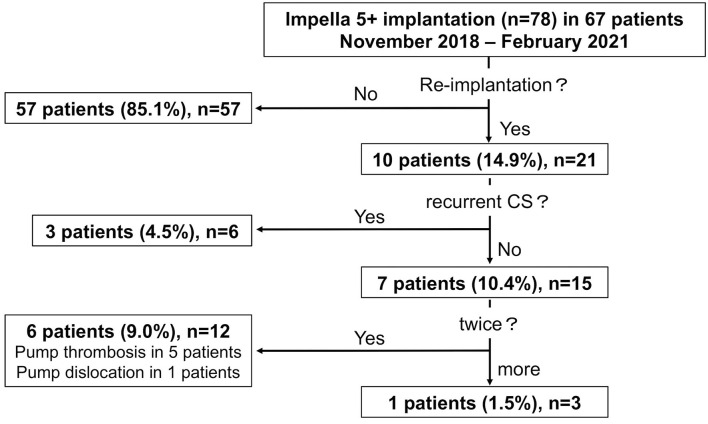
The graphic flow chart of study population. Seventy-eight Impella 5+ (5.0 or 5.5) implantation in 67 patients. CS, cardiogenic shock.

In consecutive 67 patients, a total of 78 Impella 5+ were implanted between November 2018 and February 2021 at our department, and all the cases were assigned to this study. Fifty-seven patients underwent Impella 5+ implantation as a single therapy action, whereas 10 patients (14.9%) received mechanical circulatory support *via* Impella 5+ more than one time (re-implantation). Among 10 patients with re-implantation of Impella 5+, 3 patients received the second Impella 5+ because of recurrent CS after an initial successful weaning of the first Impella 5+ and discharge from our department within the observation period. Besides, seven patients (10.4%) needed an exchange of Impella 5+ urgently due to Impella dysfunction, one of whom (1.5%) required an exchange of Impella 5+ two times (Figure 1).

### Definition of Clinical Outcomes

Regarding clinical outcomes, we investigated whether the patient could be discharged from the specialized cardiac unit at the university hospital (cardiac surgery or cardiology department). For example, if the patient was transferred to other facilities only for rehabilitation purposes, such transfer was considered “discharge”. According to this definition, overall survival considers patients after Impella 5+ support who could be discharged from the specialized cardiac unit. On the contrary, in-hospital mortality was defined as death without discharge from the cardiac unit. Besides, 30-day survival was defined as survival at 30 days after the first Impella 5+ implantation.

Further, the analysis of clinical outcomes was centered on the individual patients and not on each implanted Impella 5+ device. It means that only clinical outcomes since the last Impella 5+ support were analyzed in patients who required more than one Impella 5+ support to avoid artifacts in reported clinical outcomes. Furthermore, major bleeding was defined as prolonged or excessive bleeding, which was severe to control conservatively. It occurred spontaneously or following a medical maneuver, e.g., cardiopulmonary resuscitation (CPR) or surgical treatment. “Impella dysfunction” contains pump dislocation and pump thrombosis in this study. “Leg ischemia” means the leg hypoperfusion at the site where Impella 5+ was implanted.

### Preoperative Assessment and Surgical Procedure

A CT angiography was performed only when feasible because of the constellation of patients with CS for preoperative assessment of the whole vascular systems, e.g., aorta, subclavian artery (SA), and femoral artery (FA). In principle, at our institution SA is the standard access route for implantation of Impella 5+, allowing early patient extubation and mobilization. As an aspect of hygiene control, the SA approach is considered to be superior to the FA approach. Thus, Impella 5+ was inserted through FA only when SA access was impractical or for some other apparent reason. No matter which approach of Impella 5+ implantation was selected, all patients received Impella 5+ pump over a 10-mm Dacron prosthesis (Gelweave, Vascutek, Terumo; Renfrewshire, Scotland) implanted in an end-to-side fashion onto the target artery (so-called chimney configuration). The prosthesis was led out through the skin *via* another additional incision. Insertion and final positioning were controlled using fluoroscopy combined with transesophageal echocardiography.

Concerning the surgical procedure of Impella 5+ explantation, we clamped the prosthesis after removing Impella 5+ catheter. After flushing thrombotic materials, we cut down the prosthesis leaving a 10–15 mm long stump, which was sewed over for closure. On the other hand, regarding the procedure of Impella re-implantation, in general, the proximal and distal side of the target site with the stump of the previous prosthesis was clamped after adequate heparin administration (activated clotting time; ACT > 200 s). The remnant prosthesis was re-opened and then the included thrombotic materials were removed. A new 10-mm Dacron prosthesis was anastomosed on the stump of the previous prosthesis (end-to-end anastomosis). Of note, all prostheses implanted onto the target arteries were gelatin-coated and incubated with Rifampicin (600 mg) for minimum of 5 min before use.

### Postoperative Management of Impella 5+

Postoperative management after Impella 5+ follows as we described before (9, 10). Briefly, all parameters displayed in Impella monitor, e.g., Impella setting, Impella flow, purge pressure, purge flow rate, and placement signal, were documented every 3 h. All patients underwent chest X-ray evaluation every day to examine the position of the Impella pump. Transthoracic echocardiographic (TTE) evaluation was performed if Impella position in chest X-ray was moved or at the timing of routine TTE check-up for cardiac functions or in case of abnormal sign of Impella parameters. The anticoagulation for Impella was administrated according to the current recommendation of the manufacturer. As far as anticoagulation for purge solution is concerned, 5% dextrose in water with heparin (50 U/ml) was used as the standard.

Moreover, systemic administration of unfractionated heparin was also provided for optimal anticoagulation, with aPTT (activated partial thromboplastin time) monitored every 8 h until values became stable over 45 se. However, both anticoagulation agents were regulated appropriately in case of significant bleeding, i.e., major bleeding. In the case of heparin-induced thrombocytopeniaII, argatroban was administrated instead of heparin (10). For the evaluation of whole organs, blood gas analysis was routinely performed several times per day. The blood sampling test was also done every day.

### Weaning Strategy of Temporary MCS

Weaning of temporary MCS was conducted in those patients who experienced a minimal level of cardiopulmonary recovery. The latter level of cardiopulmonary recovery was defined as cardiac index (CI) ≥2.2 l/min with a stable or less than moderate catecholamine support and an improved organ perfusion, declining core laboratory parameters (i.e., lactate, transaminase, and creatinine). In the setting of ECMELLA, generally the weaning of venous-arterial extracorporeal membrane oxygenation (va-ECMO) was prioritized over that of Impella. However, we decided an individual weaning strategy in a case-by-case manner, defining which device would be weaned first. As far as a transition to permanent MCS is concerned, we performed LVAD implantation or oHTX when 1) temporary MCS could not be weaned off, and 2) the patients were not too old (no limitation for LVAD implantation as destination therapy (our maximal age for LVAD recipients in this period was 76 years old), oHTX until 65 years), after exclusion of major neurological deficit using computed tomography (CT) and with prior informed consent given by the patients and/or their family.

The weaning of Impella 5+ was performed by gradually reducing the Impella flow setting until P2. Then, explantation of Impella 5+ was performed if patient remained hemodynamically stable. The decision of re-implantation vs. simple explantation in the setting of Impella dysfunction (i.e., exchange of Impella device) was made based on the whole clinical situation, such as persisting inappropriate hemodynamic status (CI < 2.2 l/min, more than moderate catecholamine support, missing LV ejection) following an interdisciplinary discussion.

### Statistical Analysis

The statistical analyses were administrated with the Statistical Package for Social Sciences® (SPSS) 25.0 (IBM, Chicago, USA). Using this program, descriptive and comparative statistics were performed. Chi-Quadrat-Test and Odds Ratio (OR) were conducted for nominally scaled variables. However, Fisher's exact test was adapted instead of Chi-Quadrat-Test for expected values of <5. *P* < 0.05 were considered statistically significant.

## Results

### Clinical Outcome of Impella 5+

**Figure 2:**
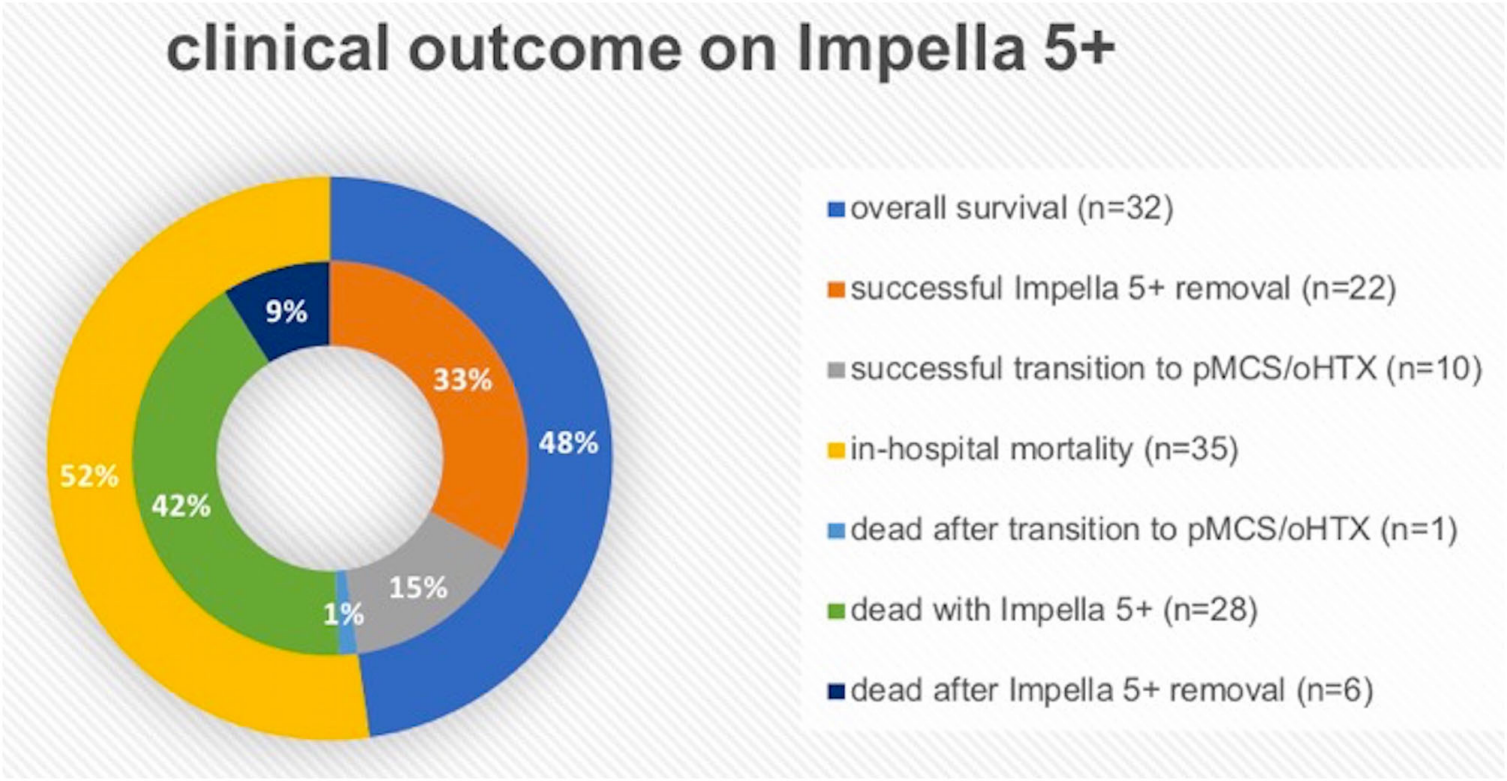
The graphic explanation of clinical outcome on Impella 5+ (5.0 or 5.5). oHTX, orthotopic heart transplantation; pMCS, permanent mechanical circulatory support.

Table 1 shows the baseline clinical characteristics of 67 consecutive patients enrolled in this study. The majority of patients were male (*n* = 56, 86.6%) with a mean age of 61.2 ± 11.4 years at Impella 5+ implantation. Acute coronary syndrome/ischemic cardiomyopathy (*n* = 55, 82.1%) is the most common underlying disease for acute CS, followed by decompensation due to dilated cardiomyopathy (DCM; *n* = 9, 13.4%). Adverse events associated with Impella 5+ support in all 67 patients are shown in Table 2. The 30-day survival was 55.2% (*n* = 37), of whom 5 patients were deceased in-hospital in the later course on postoperative day (POD) 32, 48 POD, 73 POD, 103 POD, and 210 POD, respectively. Thus, overall survival was 47.8% (*n* = 32), whereas in-hospital mortality was 52.2% (*n* = 35). We removed Impella 5+ from 39 patients (58.2%), of whom 22 patients (32.8%) recovered without permanent MCS, and 10 further patients (14.9%) survived after a successful transition to permanent MCS. On the other hand, 6 patients (9.0%) deceased after Impella 5+ removal, and 1 patient (1.5%) died from septic shock 102 days after combined oHTX and kidney transplantation (Figure 2). Interestingly, we found surgical site infection (SSI) only in this patient (1.5%), in whom revision surgery became necessary to remove the remnant Dacron prosthesis. Indeed, this patient underwent Impella 5.0 re-implantation before oHTX. Thus, SSI was found in 10% of patients who underwent re-Implantation of Impella 5+ (*n* = 1/10), whereas we found no SSI in patients with primary Impella support (0%; *n* = 0/57). Notably, an embolic event of the right arm after the removal of Impella 5+ *via* SA was observed in 3 patients (9.1%, among 33 patients who underwent the removal of Impella 5+ *via* SA), whereas 33.3% of patients with femoral Impella 5+ (*n* = 2, among 6 patients who underwent Impella 5+ implantation *via* FA) suffered from leg ischemia. Therapy withdrawal was performed due to cerebral vascular accidents (CVA) in 7 patients (19.4% of all mortality).

**Table 1:** Baseline clinical characteristics.

	**All patients**		**Re-Impella patients**	
	**(*n* = 67)**		**(*n* = 10)**	
Age (y)	61.2 ± 11.4		58.3 ± 8.49	
Male, n (%)	58	(86.6)	7	(70.0)
INTERMACS profiles I, n (%)	32	(47.8)	4	(40.0)
Arterial hypertension, n (%)	40	(59.7)	2	(20.0)
Hyperlipidemia, n (%)	16	(23.9)	1	(10.0)
Diabetes, n (%)	22	(32.8)	3	(30.0)
Peripheral vascular disease, n (%)	6	(9.0)	0	(0.0)
Arrhythmia, n (%)	23	(34.3)	5	(50.0)
COPD, n (%)	5	(7.5)	1	(10.0)
Nicotine abuses, n (%)	22	(32.8)	3	(30.0)
Drug abuses, n (%)	2	(3.0)	0	(0.0)
Dialysis, n (%)	3	(4.5)	0	(0.0)
History of PCI, n (%)	20	(29.9)	5	(50.0)
Post CPR, n (%)	18	(26.9)	5	(50.0)
Biventricular failure, n (%)	38	(56.7)	4	(40.0)
ACS/ICM, n (%)	55	(82.1)	7	(70.0)
DCM, n (%)	9	(13.4)	3	(30.0)
Myocarditis, n (%)	2	(3.0)	0	(0.0)
CS after oHTX, n (%)	1	(1.5)	0	(0.0)
va-ECMO implantation, n (%)	47	(70.1)	6	(60.0)

*Data documented as n (%) or mean ± standard deviation*.

*ACS, acute coronary syndrome; COPD, chronic obstructive pulmonary disease; CPR, cardiopulmonary resuscitation; CS, cardiogenic shock; DCM, dilated cardiomyopathy; ICM, ischemic cardiomyopathy; INTERMACS, interagency registry for mechanically assisted circulatory support; oHTX, orthotopic heart transplantation; PCI, percutaneous coronary intervention; va-ECMO, venous-arterial extracorporeal membrane oxygenation*.

**Table 2:** Clinical outcomes of Impella 5+ support focusing on adverse events.

**Patients**		**All (*n* = 67)**		**ECMELLA (*n* = 47)**		**Solo Impella (*n* = 20)**		***p***
30-day survival, n (%)		37	(55.2)	22	(46.8)	15	(75.0)	0.06
In-hospital mortality, n (%)		35	(52.2)	27	(57.4)	8	(40.0)	0.29
	MOF	25	(72.2^*^)	21	(77.8^*^)	5	(62.5^*^)	
	CVA	7	(19.4^*^)	4	(14.8^*^)	3	(37.5^*^)	
	SS/SIRS	3	(8.3^*^)	2	(7.4^*^)	1	(12.5^*^)	
HIT II, n (%)		6	(9.0)					
SSI, n (%)	Axillary access	1	(1.5)					
	Femoral access	0	(0.0)					
Arm embolism after removal, n (%)	Axillary access	3	(9.1^**^)	1	(3.0^**^)	2	(6.1^**^)	
Leg ischemia during support, n (%)	Femoral access	2	(33.3^***^)	2	(33.3^***^)	0	(0.0^***^)	
Re-implantation of Impella 5+, n (%)		10	(14.9)	7	(14.9)	3	(15.0)	

*Data documented as n (%)*.

*CVA, cerebral vascular accident; ECMELLA, venous-arterial extracorporeal membrane oxygenation + Impella; HIT, heparin-induced thrombocytopenia; MOF, multiple organ failure; SIRS, systemic inflammatory response syndrome; SS, septic shock; SSI, surgical site infection*.

* *, % of all mortality in each group;*

** *, % among 33 patients who underwent the removal of axillary Impella 5+;*

*** *, % among 6 patients who underwent Impella 5+ implantation via femoral*.

### Femoral Access for Impella 5+

As described, Impella 5+ implantation was performed *via* FA only as a second choice if implantation *via* SA was deemed unfavorable, which is in line with current practice globally (11). In this sense, Impella 5+ was implanted *via* FA only in 6 patients (9.0%) (Table 3). In four patients (66.7%), the implantation was converted to FA access intraoperatively. In one patient, the exchange from Impella CP to 5.0 was performed *via* the same FA. In contrast, in another patient, the implantation was performed *via* the same side of FA after open repair of FA due to massive bleeding after the attempt of punctual arterial cannulation of va-ECMO. In two patients with concomitant va-ECMO support simultaneous to FA Impella 5+, an antegrade reperfusion cannula directed to the distal leg was inserted on the side of Impella due to suspected leg ischemia (33.3%, *n* = 2). Interestingly, the mortality of the femoral Impella 5+ was tendentially higher as the group of Impella 5+ implantation *via* SA in all cohorts (FA vs. SA, 83.3% (*n* = 5/6) vs. 49.2% (*n* = 30/61), *p* = 0.20). Among only 47 ECMELLA patients, this tendency also remains (FA vs. SA, 83.3% (*n* = 5/6) vs. 41.5% (*n* = 22/41), *p* = 0.22) (Table 4).

**Table 3:** Patients profile of Impella 5+ implantation *via* the femoral artery.

**Age (y)**	**Sex**	**Height (m)**	**Weight (kg)**	**Va-ECMO?**	**Impella size**	**Why not *via* SA?**	**Diameter of SA (mm)**		**Leg ischemia?**	**Re-implantation?**	***Via* FA again?**	**Clinical outcome**
							**Pre. CT**	**Post. CT**				
61.8	F	1.57	61	Yes	5.0	Inadequate diameter of SA	-	4.0	No	No	-	Living
60.1	M	1.74	70	Yes	5.0	Inadequate diameter of SA	-	4.9	Yes	No	-	Deceased
41.2	M	1.75	110	Yes	5.0	Inadequate diameter of SA	4.5	8.0	No	No	-	Deceased
40.7	M	1.85	100	Yes	5.0	Inadequate diameter of SA	5.8	-	Yes	No	-	Deceased
49.6	F	1.58	92	Yes	5.0	Impella change from CP to 5.0	-	-	No	Yes	Yes	Deceased
68.0	M	-	90	Yes	5.0	Opened groin; FA repair due to inguinal hemmorrage after emergent va-ECMO attempt	-	-	No	Yes	Yes	Deceased

*CT, computed tomography; d, days; F, female; FA, femoral artery; M, male; post., postoperative; pre., preoperative; SA, subclavian artery; va-ECMO, venous-arterial extracorporeal membrane oxygenation; y, years; -, no available data or unnecessary information*.

**Table 4:** Representative outcomes in 47 ECMELLA patients depending on access site.

**ECMELLA patients**	**All**		**Femoral access**		**Axillary access**		**p**
	**(*n* = 47)**		**(*n* = 6)**		**(*n* = 41)**		
Impella dysfunction, n (%)	5	(10.6)	3	(42.8*)	3	(7.3)	0.03
In-hospital mortality, n (%)	27	(57.4)	5	(83.3*)	22	(53.7)	0.22

Data documented as n (%). ECMELLA, va-ECMO, venous-arterial extracorporeal membrane oxygenation + Impella; ^*^, n (%) of 6 femoral Impella 5.0/5.5 + 1 femoral Impella CP.

### Re-implantation of Impella 5+

Characteristics of patients undergoing re-implantation of Impella 5+ are presented in Table 5. A re-implantation of Impella 5+ was necessary in a total of 10 patients due to (1) recurrent CS (*n* = 3), (2) Impella thrombosis (*n* = 5), and (3) Impella dislocation (*n* = 2). Additionally, we observed 2 further patients with a change of left ventricular unloading therapy involving one Impella 5+, where in one patient LVAD implantation was performed as a direct transition after dislocation of Impella 5.0 (patient suppl. 1 in Table 5). In another patient (patient suppl. 2 in Table 5), Impella 5+ was upgraded from Impella CP due to the dislocation of Impella CP inserted *via* FA. Concerning Impella dysfunction including one Impella CP patient (patient suppl. 2 in Table 5), the FA access was a significant risk factor for Impella dysfunction compared to the SA access (FA vs. SA, 42.8% (*n* = 3/7) vs. 9.8% (*n* = 6/61), *p* = 0.04, OR 6.88, CI 1.23–38.3). This evidence was also found in the setting of ECMELLA (FA vs. SA, 42.8% (*n* = 3/7) vs. 7.3% (*n* = 3/41), *p* = 0.03, OR 9.5, CI 1.42-63.7). On the other hand, no statistical difference of overall mortality was observed in patients with Impella 5+ re-implantation (*n* = 10) compared to patients with primary Impella 5+ support (*n* = 57) (mortality 80.0% (*n* = 8/10) for ECMELLA setting vs. 47.4% (*n* = 27/57) for primary Impella setting, *p* = 0.09) (Table 4).

**Table 5:** Re-implantation of Impella 5+ in 10 patients.

**Patient**	**Age (y)**	**Sex**	**Size of prev. Impella**	**Duration of prev. Impella (d)**	**Why re-implantation?**	**Window period (d)**	**Size of new Impella**	**Impella access site**		***Via* prev. vasc. graft?**	**Remove prev. graft?**	**New graft on prev. graft?**	**Clinical outcome**	**Cause of death**
								**Prev**.	**New**					
1	72.4	F	5.0	11	Recurrent CS	6	5.0	SA	SA	No	No	Yes	Deceased	CVA
2	54.9	M	5.0	8	Recurrent CS	13	5.0	SA	FA	-	-	-	Deceased	CVA
3	52.5	M	5.0	4	Recurrent CS	203	5.0	SA	SA	No	No	Yes	Living atter oHTX	-
4	49.6	F	5.0	13	Pump thrombosis	-	5.0	FA	FA	Yes	No	No	Deceased	SS
5	64.1	M	5.0	5	Pump thrombosis	-	5.0	SA	SA	No	No	Yes	Deceased	SS
6	60.7	M	5.0	27	Pump thrombosis	-	5.0	SA	SA	No	No	Yes	Living after LVAD	-
7	59.8	M	5.0	26	Pump thrombosis	-	5.5	SA	SA	No	No	Yes	Deceased After oHTX	SS
8	56.5	M	5.5	13	Pump thrombosis	-	5.0	SA	SA	No	No	Yes	Deceased	MOF
9	68.0	M	5.0	5	Pump dislocation	-	5.0	FA	FA	No	No	Yes	Deceased	MOF
10	44.5	F	5.0	39	Upgrade of Impella	-	5.5	SA	SA	No	Yes	No	Deceased	CVA
			5.5	28	Pump dislocation	-	5.0	SA	SA	No	No	Yes		
Suppl. 1	37.4	M	5.5	6	Pump dislocation	-	LVAD	SA	-	-	-	-	Living after LVAD	-
Suppl. 2	34.5	M	CP	1	Pump dislocation	-	5.0	FA	SA	-	-	-	Living afer oHTX	-

*CP, Impella CP; CS, cardiogenic shock; CVA, cerebral vascular accident; d, days; F, female; FA, femoral artery; LVAD, left ventricular assist device; M, male; prev., previous; MOF, multiple organ failure; oHTX, orthotopic heart transplantation; SA, subclavian artery; SS, septic shock; suppl., supplemental; vasc., vascular; y, years; -, no available data or unnecessary information; 5+, 5.0 or 5.5*.

In all cases, we did not find complications associated with re-exploration of the artery or Impella re-implantation. As far as the operative technique of re-implantation of Impella 5+ is concerned, we used a new prosthesis as described above in nine patients. Only for one patient (patient 4 in Table 5), we used the index prosthesis for re-implantation *via* FA after removing thrombosis materials. Further, in one case (patient 10 in Table 5), the entire prosthesis used for previous Impella implantation was removed entirely before anastomosis of a new prosthesis.

### Pump Thrombosis of Impella 5+

Details of patients with pump thrombosis are presented in Table 6. One patient (Case 6) did not receive an exchange of Impella due to adequate hemodynamics under ongoing va-ECMO support, and he recovered fully without permanent MCS in the further course. Thus, the prevalence of pump thrombosis was 9.0%, with 15.7 ± 8.89 days of mean support duration in this cohort. The patients of case 1 and case 4 had clinically severe major bleeding (thoracic bleeding due to status post CPR and massive inguinal re-bleeding post-va-ECMO in case 1, bladder bleeding in case 4) so that the purge anticoagulation was changed to low dose heparin (heparin 20 U/ml) in the last few days, and systemic anticoagulation was ceased. In all cases, thrombus mass was observed as an obvious finding within the Impella pump at the time of explantation.

**Table 6:** Clinical characteristics of 6 patients at the timing of pump thrombosis.

	**Case 1**	**Case 2**	**Case 3**	**Case 4**	**Case 5**	**Case 6**
**Patient Nr. in Table 3**	**Patient 4**	**Patient 5**	**Patient 6**	**Patient 7**	**Patient 8**	
Basis diagnosis	ACS (4 year after oHTX)	DCM	ACS	ICM	DCM	Myocarditis
Impella size	5.0	5.0	5.0	5.0	5.5	5.0
Impella duration (d)	13	5	27	26	13	10
Va-ECMO ?	Termimated	No	Terminated	No	Yes	Yes
Other system ?	TandemHeart	No	No	No	No	No
Discharged ?	Yes	Yes	No	Yes	No	No
Antiplatelet medication ?	No	No	Aspirin + Clopidogrel	No	No	No
Purge anticoagulation	D5W+ heparin 20 U/ml	D5W+ heparin 50 U/ml	D5W+ heparin 50 U/ml	D5W+ heparin 20 U/ml	D5W+ heparin 50 U/ml	D5W+ argatroban 0.09 mg/ml
aPTT within 24h (sec)	27	48	70	30	79	30
INR within 24h	1.1	1.5	2.1	1.1	1.2	1.3
Hit II	Negative	Negative	Negative	Negative	Negative	Positive
Platelets (×1000 μl)	85	119	216	98	130	75
Impella setting	P4	Pump stop	P7	P8	P2	P3
Impella flow (l/min)	Transient immeasurable		4.1	4.2	1.0	0.7
Purge flow rate (ml/h)	14.0		21.6	2.3	5.0	<1
Purge pressure (mmhg)	396		543	972	639	1065

*ACS, acute coronary syndrome; aPTT, activated partial thromboplastin time; d, days; DCM, dilatative cardiomyopathy; D5W, dextrose 5% in water; h, hour(s); HIT, heparin-induced thrombocytopenia; ICM, ischemic cardiomyopathy; INR, international normalized ratio; min, minute; oHTX, orthotopic heart transplantation; sec, seconds; U, units; va-ECMO, venous-arterial extracorporeal membrane oxygenation; -, no available data or unnecessary information*.

## Discussion

Although Impella has already been widely utilized for various CS situations, few reports focus on postoperative adverse events (1, 3–6). Further, clinical outcomes of Impella 5+ re-implantation have not been reported.

The key findings of this observational retrospective study are: (1) 15% re-implantation rate with 48% overall survival; (2) 9.0% incidence of pump thrombosis; (3) no complications of re-implantation procedure and a low incidence SSI when using rifampicin-incubated gelatin-coated Dacron prosthesis; (4) considerable morbidity (9.1 %) of arm embolism after the removal of Impella 5+ *via* SA and finally; (5) statistically significantly higher Impella dysfunction rate (p = 0.04, OR 6.88) with numerically higher mortality in the FA access sub-cohort.

In our study, pump thrombosis was the main indication for re-implantation of Impella 5+ (50%. *n* = 5/10). We do not know whether this result reflects a common range to be expected for Impella 5+ because the re-implantation rate and rate of pump thrombosis have not been well studied previously. However, according to our results, we can explain certain trends, which might affect the risk of pump thrombosis. As we know, the axial pump of the Impella system requires heparinized purge solution, which prevents blood from entering the motor as it flows through the Impella catheter. Besides, systemic anticoagulation to achieve therapeutic aPTT levels is recommended (7, 8).

Nevertheless, we often face the situation of restricting anticoagulation because of severe major bleeding, especially in patients with CS. In this sense, systemic anticoagulation, even anticoagulation administered *via* purge solution for Impella patients, sometimes becomes challenging. Depending on individual cases, we ought to decide on the ideal anticoagulation therapy. Previous studies underline the value of optimal anticoagulation therapy in a balance of prevention of thrombosis and the adverse result of major bleeding, respectively (1, 12–16). Despite the manufacturer's recommendation of anticoagulation management using ACT, systemic anticoagulation has been monitored by aPTT in the majority of past studies (aPTT > 45 s).

On the contrary, regarding anticoagulation of the purge solution, a reduction down to half of the heparin concentration (heparin 25 U/ml) has been described to result in a favorable outcome with no significant rise in thrombotic events (12). In our case series, we also administered less than half of the heparin concentration recommended for purge solution (20 U/ml) for one patient who unfortunately received pump thrombosis. Indeed, this patient had no systemic anticoagulation due to massive hematuria. Certainly, in such a case with purge solution's anticoagulation reduced to levels under the recommended therapeutic level. the Impella system parameters should be monitored closely.

It has been proposed that the longer the Impella support period, the higher the risk for thrombotic event rate due to the artificial profile. The presented data demonstrated a trend supporting this hypothesis. We performed an exchange of Impella 5+ in all patients with pump thrombosis. The utilization of tissue plasminogen activator (tPA) in the purge solution (0.04 mg/ml) has been recently introduced as an alternative to heparin (17, 18). In our opinion, tPA is a valid therapy option for patients with diagnosed or suspected pump thrombosis, although a decision for initiation of tPA should be made cautiously and in an individual case-by-case fashion due to the strong fibrinolytic effect of tPA.

Regarding the dislocation of Impella 5+, we encountered three cases (4.5%) in our study cohort. Only one patient also received va-ECMO support, whereas two patients had no other MCS. Thus, re-Impella or direct LVAD implantation had to be emergently performed. Bernhardt *et al*. had reported the first experience with Impella 5.5 in Germany. They reported the incidence of dislocation in 21.7% of patients in the first generation of the device with a shorter cannula (11). Since the device length has been modified, the incidence of dislocation would be improved. Although the dislocation of Impella may represent a critical situation, it is challenging to re-insert the Impella catheter through the aortic valve without a guidewire. One interesting method employing rapid ventricular pacing has been suggested to let the aortic valve “open” (19). This technique enables repositioning the Impella catheter at the “bedside,” and it would be one of the most considerable merits for patients who are at risk for hemodynamic deterioration after Impella dislocation. However, further follow-up studies are warranted.

The surgical procedure for Impella 5 + re-implantation has not yet been thoroughly discussed. Excluding one case of femoral Impella 5+, we strictly utilized a new prosthesis, so that thrombus materials in the index prosthesis could be largely removed. Given our clinical results of no complications in the re-implantation, this concept seems to be favorable. Regarding the incidence of SSI and prosthesis-associated infection, an 1.5% incidence appears to be well tolerated with the consideration of complex settings in patients with CS. Thus, we conclude that the herein applied surgical strategy for re-implantation of Impella 5+ may be used as a standardized strategy. However, it remains unclear whether rifampicin incubation is a causative contributor to the favorable outcome in terms of infection prevention.

With respect to arm embolism after removal of axillary Impella 5+, we regard this morbidity as an unacceptable one, although we observed no CVA that was directly associated with the explantation procedure. As a matter of fact, the Impella pump has been sometimes explanted without clamping of the distal artery in our previous series. Since we observed distal embolism after removing Impella in a few patients, we modified our standard procedure; recently, we clamped distal SA for embolic protection and sometimes performed the prophylactical thrombectomy using a Fogarty catheter. This technique was been already reported by Kawabori et al. (20). We regard this technique as a reasonable approach for the safe removal of Impella catheter.

In this cohort, the adverse outcomes of the femoral approach for Impella 5+ were significant. As we performed Impella 5+ implantation *via* FA as the second option, a direct comparison of the two access routes is not possible. However, our inferior outcomes of femoral Impella 5+, i.e., high incidence of leg ischemia, numerically higher mortality, and statistically higher morbidity of Impella dysfunction give us a word of caution and warrants high levels of awareness for early signs of complications when managing patients with femoral Impella 5+ support.

## Conclusion

Our results suggest relevant rate of re-implantation (14.9%, *n* = 10) and considerable prevalence of pump thrombosis (9.0%, *n* = 6) in patients receiving Impella 5+. Re-implantation was safely feasible *via* the same access route. Particular attention is warranted regarding some complications, especially in FA access with potential impact on overall mortality.

## Limitations

There are several limitations to this study. First, this manuscript deals with a retrospective observational study with a limited cohort size of non-randomized patients at a single center. Potential systematic measurement errors can affect the outcomes. Secondly, because of the limited cohort size in this study, our patients were analyzed as homogeneous patients with CS with no regard for baseline patients' characteristics and therapy variety, despite patients' heterogeneous backgrounds. Generally, the descriptive analysis of target patients is necessary to analyze more details of the study's main purpose. Further, long-term outcomes are missing in this study. We should evaluate patients with CS in the long observation period, which can provide us further insights into the clinical outcomes associated with Impella 5+ support.

## Data Availability Statement

The original contributions presented in the study are included in the article/supplementary material, further inquiries can be directed to the corresponding author/s.

## Ethics Statement

The Ethic Committee in University Hospital Düsseldorf approved this retrospective study (Ref.2020-1173). Written informed consent for participation was not required for this study in accordance with the national legislation and the institutional requirements.

## Author Contributions

Conceptualization and validation: YS and PA. Methodology, software, formal analysis, investigation, resources, data curation, writing—original draft preparation, and visualization: YS. Writing—review and editing: SB, MI, AM, PR, RW, HA, UB, AL, and PA. Supervision and project administration: AL and PA. All authors have read and agreed to the published version of the manuscript.

## Conflict of Interest

AM and PA have received speaker honoraria from Abiomed. The remaining authors declare that the research was conducted in the absence of any commercial or financial relationships that could be construed as a potential conflict of interest.

## Publisher's Note

All claims expressed in this article are solely those of the authors and do not necessarily represent those of their affiliated organizations, or those of the publisher, the editors and the reviewers. Any product that may be evaluated in this article, or claim that may be made by its manufacturer, is not guaranteed or endorsed by the publisher.
